# EFFECT OF SELF-PACED WALKING ON REDUCING COGNITIVE DEMANDS OF WALKING WHILE TALKING

**DOI:** 10.1093/geroni/igz038.2429

**Published:** 2019-11-08

**Authors:** Hyeon Jung Kim, Farahnaz Fallahtafti, Jennifer M Yentes, Dawn Venema, Julie Blaskewicz Boron

**Affiliations:** 1 University of Nebraska Omaha, Omaha, Nebraska, United States; 2 University of Nebraska at Omaha, Omaha, Nebraska, United States; 3 University of Nebraska Medical Center, Omaha, Nebraska, United States

## Abstract

Although walking is considered an automated process, internal and external factors can alter gait performance. Fear of falling leads to walking with a wider base of support and decreased walking speed, due to a combination of shorter stride lengths and/or stride time. Exacerbation of gait deficits have been reported under high cognitive load situations (HCLS, also known as dual-tasks). Talking on a phone is considered an increased HCLS over talking in-person due to visualization of the individual on the phone. The purpose of the study was to explore the added effects of walking while talking on a phone compared to talking in-person. Fifteen healthy older adult subjects (70.86±4.7yrs) performed three conditions while walking on a self-paced treadmill for ten minutes: (1)walking alone, (2)walking while talking in-person, (3)walking while talking on a phone. Mean stride length(SL), stride time(ST), and step width(SW) were compared using one-way, repeated-measures ANOVAs (p=0.10). Dual-task cost of walking while talking in-person and on a phone was calculated for each gait variable and compared with t-tests. Mean gait variables did not differ between conditions (SL p=0.95, ST p=0.77, SW p=0.57). Dual-task costs were not significantly different between talking conditions (SL p=0.99, ST p=0.54, SW p=0.14). Use of a self-paced treadmill allowed the subjects to perform in their “comfort zone”, however, walking on a set-speed treadmill may force walking speed outside of the comfort zone, pushing one’s “reserve”, and revealing differences. Use of a self-paced treadmill better approximates daily life by providing the opportunity to make adaptations under HCLS.

